# Evaluating the predictive accuracy of ion-channel models using data from multiple experimental designs

**DOI:** 10.1098/rsta.2024.0211

**Published:** 2025-03-13

**Authors:** Joseph G. Shuttleworth, Chon Lok Lei, Monique J. Windley, Adam P. Hill, Simon P. Preston, Gary R. Mirams

**Affiliations:** ^1^ Centre for Mathematical Medicine & Biology, School of Mathematical Sciences, University of Nottingham, Nottingham NG7 2RD, UK; ^2^ Institute of Translational Medicine, Faculty of Health Sciences, University of Macau, Macau, People’s Republic of China; ^3^ Department of Biomedical Sciences, Faculty of Health Sciences, University of Macau, Macau, People’s Republic of China; ^4^ Computational Cardiology Laboratory, Victor Chang Cardiac Research Institute, Darlinghurst, New South Wales, Australia; ^5^ School of Clinical Medicine, Facility of Medicine and Health, University of New South Wales, Sydney, New South Wales, Australia

**Keywords:** hERG, mathematical model, electrophysiology, optimization, validation

## Abstract

Mathematical models are increasingly being relied upon to provide quantitatively accurate predictions of cardiac electrophysiology. Many such models concern the behaviour of particular subcellular components (namely, ion channels) which, together, allow the propagation of electrical signals through heart-muscle tissue; that is, the firing of action potentials. In particular, I_Kr_, a voltage-sensitive potassium ion-channel current, is of interest owing to the central pore of its primary protein having a propensity to blockage by various small molecules. We use newly collected data obtained from an ensemble of voltage-clamp experiment designs (protocols) to validate the predictive accuracy of various dynamical models of I_Kr_. To do this, we fit models to each protocol individually and quantify the error in the resultant model predictions for other protocols. This allows the comparison of predictive accuracy for I_Kr_ models under a diverse collection of previously unexplored dynamics. Our results highlight heterogeneity between parameter estimates obtained from different cells, suggesting the presence of latent effects not yet accounted for in our models. This heterogeneity has a significant effect on our parameter estimates and suggests routes for model improvement.

This article is part of the theme issue ‘Uncertainty quantification for healthcare and biological systems (Part 1)’.

## 1. Introduction

Present throughout the human body, ion channels are protein structures embedded in the cell membrane, which play an important role in the transmission and reception of electrical signals. This is especially true of the production of cellular action potentials in heart-muscle cells (cardiomyocytes) [1]. Ion channels mediate the flow of specific species of ions into and out of the cell. The focus of this paper, K_V_ 11.1, is a potassium ion channel that opens and closes in response to voltage signals (that is, a voltage-sensitive potassium ion channel). In heart-muscle cells (cardiomyocytes) there are a large number of K_V_ 11.1 channels, through which a combined current flows (known as the *rapid delayed rectifier potassium current* and denoted by I_Kr_ [2]). The blocking of this current by small molecules is associated with dangerous changes to the heart’s rhythm (arrhythmia) [3]. Consequently, K_V_ 11.1 is a key focus of drug safety assays [4]. For this purpose, accurate mathematical models of the baseline behaviour of I_Kr_ (among other ion-channel currents) are desirable, allowing the effect of drug-channel interactions to be quantified and used to classify proarrhythmic risk [5,6]. Whilst our work here is focused on hERG1a cell lines rather than cardiomyocytes, we use I_Kr_ as a shorthand for the recorded currents. We expect some differences between these hERG1a currents and those recorded from real cardiomyoctes, as it is known that channels in cardiomyoctes may be formed from combinations of hERG1a and hERG1b, which results in morphological changes to the recorded currents [7]. Nevertheless, hERG1a cell lines are used as surrogate models in drug safety studies [8]. Moreover, the methods described herein are presented such that they may be applied broadly to other macroscopic, voltage-gated ion-channel currents.

**Figure 1.**
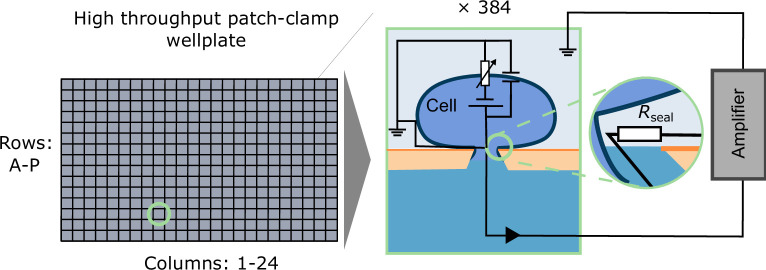
A diagram of a patch-clamp experiment performed on a high-throughput, automated patch-clamp platform. A seal is formed between the plate and the cell. Automatically applied pressure is then used to puncture the cell membrane such that an electrical current flows from the inside of the cell, through the membrane, to the amplifier where it is recorded. Each of the squares on the wellplate (left) represents a well in which the current from a single cell is recorded. Figure modified from [15].

Typically, models of macroscopic ion-channel currents are built, fitted and validated using data collected from *patch-clamp electrophysiology* experiments [9,10]. We adopt this approach in this paper, performing room-temperature, whole-cell voltage-clamp experiments on a high-throughput, automated patch-clamp platform. These experiments allow I_Kr_ to be measured whilst the transmembrane potential, $V_{\text{m}}$, is manipulated. Such experiments can produce a wealth of information-rich data [11], which may be used to fit mathematical models of macroscopic ion-channel currents [12–14]. A diagram of such an experiment is shown together with an equivalent electrical circuit in figure 1. We use the resulting data to train and validate I_Kr_ models, as described below.

While mathematical models are increasingly being used to quantify the effect that drugs have on I_Kr_ and other ion-channel currents [6], many conflicting, yet plausible, mathematical models are suggested in the literature [16,17]. Typically, these models are *Markov models* [12], including different numbers of parameters and differences in the number of states and how they are connected (the *model structure*). Regarding the choice of model structure, Mangold *et al*. enumerated many thousands of possible model structures for Markov models of the fast sodium current, $I_{\text{Na}}$ and the fast-transient outward potassium current [18]. Many of these structures may also yield plausible models of I_Kr_ [11], albeit with significantly different parameter values. Note that other models, such as the so-called Hodgkin–Huxley models, may be expressed as Markov models, even if they were not originally presented this way [12,19,20]. However, it is not clear which model (or models) provides the most accurate description of I_Kr_ [11], or which is the most suitable for use in drug-binding assays or inclusion in whole-cell action-potential models [20,21]. By simulating the dynamics of these models under various protocols, and performing extensive validation of predictive accuracy, we aim to select the most suitable model structures and, as a result, obtain more accurate predictive models of I_Kr_.

Though probabilistic models may be used to study ion channels [22] (especially for models of single channels [23]), Markov models of I_Kr_ are typically implemented deterministically as systems of ordinary differential equations (ODEs) [12]. In all the models we consider, only a single ‘open’ conformation allows current to flow through the channel, and so the I_Kr_ current is proportional to the fraction of channels in this conformation. Numerous such models of I_Kr_ are suggested in the literature, with various contradicting model structures; they disagree on the number of conformational states, which transitions between states are possible, and certain symmetries between the rates at which transitions occur [16]. Therefore, we aim to develop the methodology necessary to select the most accurate from a pool of candidate models.

Using synthetically generated data, we have previously shown that we are able to identify a correct model structure from a pool of candidates, and accurately infer model parameters, resulting in improved predictive accuracy [24]. Here, we adapt and apply this methodology to newly collected experimental data using a wider range of experimental designs. In particular, we apply a diverse range of voltage-clamp *protocols* (that is, the user-defined voltage signals that comprise our experimental design) to a selection of cells and record the resulting currents simultaneously. However, the extension of this work to real data introduces additional complications—not least of which is the fact that our approximate mathematical models are incapable of fully recapitulating the underlying *data-generating process* [25]. Nevertheless, these newly collected data allow us to compare the predictive accuracy of a collection of literature I_Kr_ models.

The variability in parameter estimates obtained by fitting models to real, experimental data from different cells has been explored previously in the literature [26]. It is unclear to what extent this is the result of underlying biological variability or other non-biological factors affecting the recorded current (experimental artefacts). As in [26], the data discussed herein were collected from an automated patch-clamp set-up where a series of voltage-clamp protocols are performed (in parallel) on 384 separate wells, each of which, in an ideal scenario, records the resultant current in a single cell. After fitting our models to these data, we consider the variability of our parameter estimates (each taken from a given well and protocol) in §6. Here, we show that our parameter estimates depend, not only on the voltage protocol used, but on the particular cell/well from which the data were obtained. This provides insight into the nature of the discrepancy between our mathematical models and the data-generating process. This analysis suggests the presence of latent well-dependent effects, which are not yet accounted for in our mathematical models.

## 2. Mathematical models of I_Kr_

Each of the four Markov models we consider is an ODE-based model with a *governing equation*,


(2.1) $$\begin{array}{rl} \frac{\text{d}}{\text{dt}}x & =Q(V_{\text{m}}{)}^{⊤}x, \end{array}$$

where x is a *state-variables* vector which describes the portion of channels in each of the model’s conformational states, Q is a voltage-dependent transition rate matrix [27] such that the element $Q_{i,j}$ is the transition rate between the model’s i^th^ and j^th^ states and $V_{\text{m}}$ is the *transmembrane potential*, that is, the potential difference between the inside and outside of the cell membrane. These states are mapped to our observables, via an *observation function* of the form


(2.2) $$\begin{array}{lrlr} & I_{\text{Kr}}(t) & =gx_{\text{O}}(t)(V_{\text{m}}(t)-E_{\text{Kr}}), & \end{array}$$

where $E_{\text{Kr}}$ is the *reversal potential*, g is the maximal conductance and $x_{\text{O}}$ is the state in the vector x representing the open channel conformation.

Typically, the model’s reversal potential, $E_{\text{Kr}}$, is set to the Nernst potential which may be calculated as


(2.3) $$\begin{array}{lcr} & E_{\text{Nernst}}=\frac{RT}{F}\ln\left\{\frac{\left[K_{\text{out}}\right]}{\left[K_{\text{in}}\right]}\right\}, & \end{array}$$

where $\left[K_{\text{out}}\right]$ denotes the extracellular potassium concentration, $\left[K_{\text{in}}\right]$ denotes the intracellular potassium concentration, R is the gas constant, F is Faraday’s constant and $T=298.15\;\text{K}=25^{\circ }\text{C}$ is the temperature at which our experiments were performed [28]. This is the transmembrane potential at which, according to the model, there is no force driving K^+^ ions through the channel (see equation (2.2)). Using the known potassium concentrations of our intracellular and extracellular solutions, (132mM and 4mM, respectively), we find $E_{\text{Nernst}}\approx -90\;\text{mV}$.

We assume that the initial state vector, x(0), lies at the governing equation’s unique global equilibrium point. Such an equilibrium point is guaranteed to exist for our choice of models [12,29]. The assumption is that the model is at equilibrium when t=0 is made because the cell is left to equilibrate before each protocol (that is, before each *sweep* is recorded). During this time, the command voltage, $V_{\text{cmd}}$ is held at the holding potential, −80mV, and we can compute the resulting steady state, which depends on the model parameters, θ [12].

Various Markov models, each characterized by a different choice of Q can be used here. Note that the number of states in the model, N, may also vary, meaning that the length of the state-variable vector, $x\in R^{N}$, may differ between models. For each model, transition rates, $Q_{i,j}$ are dictated by our model parameters. Typically, we have rates of the form $Q_{i,j}=A\;\exp\{\pm bV\}$ where A and b are model parameters (such as $p_{1}$ and $p_{2}$ in the Beattie model [11]). The Wang model [30], however, contains two voltage-independent transition rates, $k_{f}$ and $k_{b}$, which are, themselves, scalar model parameters. Also, for each model, the maximal conductance, g, is fitted as an additional model parameter, acting as a scaling factor, see equation (2.2).

**Figure 2.**
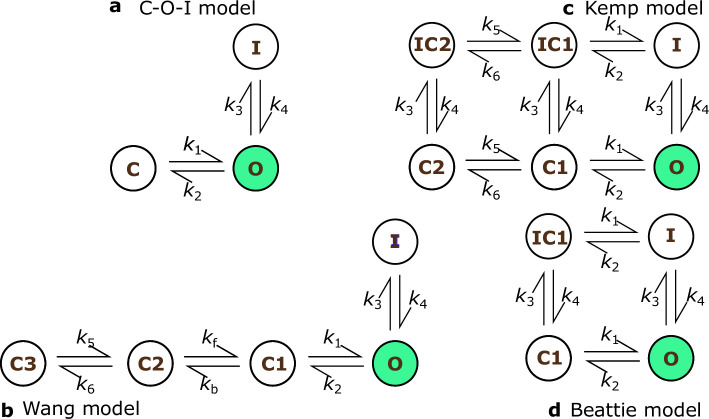
a-d indicate the four model structures used. Transition rates are parameterised by two parameters: all rates with even indices have rates of the form, $k_{2i}=A\exp\{-bV_{\text{m}}\}$ and all odd numbered rates are of the form, $k_{2i+1}=A\exp\{bV_{\text{m}}\}$, except $k_{f}$ and $k_{b}$ in the **C**1 to **C2** transition in the Wang model which are both constant rates [30].

We consider the four models shown in figure 2: the Beattie model [11]; the Kemp model [31]; the Wang model [30] and a simple, three-state model which we refer to as the closed-open-inactive (C-O-I) model. These models differ in the number of states (that is, N) and parameters (those which determine transition rates), and in the existence of a path between the inactivated state (**I**) and the closed states (**C/C1/C2/C3**) that avoids the open state, **O**. Of these models, the Wang model has the most model parameters (15 including the maximal conductance parameter, g) whereas the C-O-I and Beattie models have the fewest (nine parameters in total). However, each model shares the same form, satisfying equations (2.1) and (2.2). While the reversal potential is treated as a known constant, the maximal conductance, g, like our transition-rate parameters, is fitted independently for each individual sweep.

To fit a given model, we assume the data were generated using the given model structure with some unknown parameter set, θ. Then, we assume that our observations, $z_{i}$, are subject to additive, independent and identically distributed (IID) Gaussian errors, $\varepsilon_{i}$ such that


(2.4) $$z_{i}=y_{i}(\theta )+\varepsilon_{i},$$

where $y_{i}(\theta )$ is the observable corresponding to the i^th^ observation, which depends on the model parameters, θ. Then, we compute the maximum likelihood estimate (MLE), $\hat{\theta }$, by finding the parameter set which minimises $\sum_{i=1}^{n}(y_{i}-z_{i}{)}^{2}$, where n is the number of observations. For this model, MLE is equivalent to nonlinear least-squares regression.

**Figure 3.**
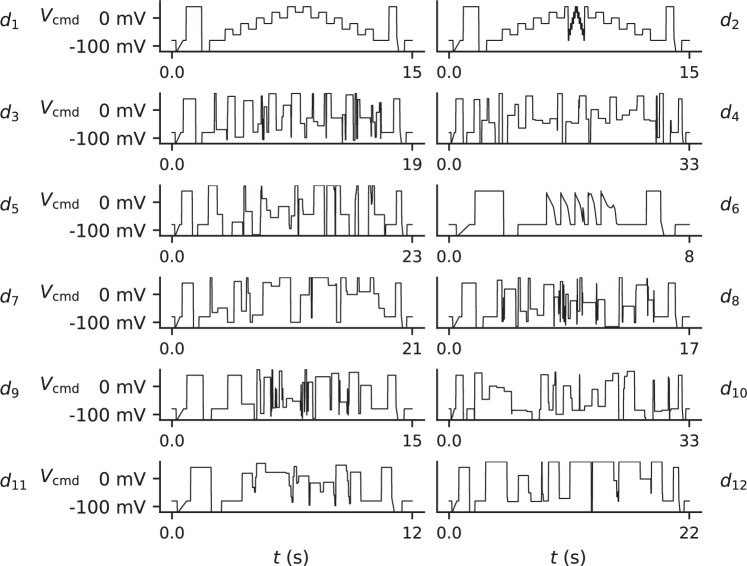
The protocols used in our experiment, shown in the order that they are applied. Common features present at the start and end of each protocol are used for postprocessing. All protocols are repeated exactly once except $d_{1}$, Lei *et al. staircase* protocol. All protocols are used for model validation except $d_{6}$, which is used only for validation.

The command voltage, $V_{\text{cmd}}(t)$ , is a time-dependent waveform which the experimenter is free to choose to apply as a voltage clamp. In this article we apply 12 protocols, **$d_{1}$** to **$d_{12}$**, as our $V_{\text{cmd}}(t)$ as shown in figure 3, with their designs discussed in §4. After computing a parameter estimate for each sweep of the protocols for each model, we use these parameter estimates to compute predictions for the remaining protocols.

We assume that our data arise from an *ideal* patch-clamp set-up. That is, we assume that at any time, t, the membrane voltage is exactly the command voltage, albeit with a possibly non-zero systematic voltage error, that is


(2.5) $$\begin{array}{lcr} & V_{\text{cmd}}(t)=V_{\text{m}}(t)+V_{\text{off}}. & \end{array}$$

This voltage-offset is included to explain the discrepancy between $E_{\text{Nernst}}$ and $E_{\text{obs}}$ as discussed in the electronic supplementary material, section D. Next, we assume that the current we observe during the experiment is exactly a combination of I_Kr_ and our linear-leak current,


(2.6) $$\begin{array}{lcr} & I_{\text{out}}=I_{\text{L}}+I_{\text{Kr}}, & \end{array}$$

where $I_{\text{L}}$ is the leak current satisfying


(2.7) $$\begin{array}{lcr} & I_{\text{L}}=g_{\text{L}}(V_{\text{cmd}}-E_{\text{L}}), & \end{array}$$

where $g_{\text{L}}$ is the leak conductance and $E_{\text{L}}$ is the reversal potential of the leak current. These two parameters are fitted during *postprocessing* (before our Markov-model parameters are fitted) as described in §5.

## 3. Experimental methods

### (a) Cell culture and harvesting

For cell harvesting, we used the same methods described in [32,33]; hERG channels stably expressed in Chinese hamster ovary (CHO) cells were purchased from the American Type Culture Collection (ATTC reference PTA−6812). The CHO cells were maintained in Hams Nutrient mix media (ThermoFisher Scientific, Waltham, USA) supplemented with 5% fetal bovine serum (Merck Life Science, Melbourne, VIC, Australia). Cells were housed in a 37°C humidified incubator at 5% CO_2_. Cells were passaged every 2−3 days and harvested for experiments 48−72 h after passaging. Prior to harvesting, cells were grown in t150 or t175 tissue culture flasks to a confluency of 60–80%. Confluent cells were washed twice with phosphate-buffered saline (PBS, Mg/Ca^2+^ free, ThermoFisher Scientific, Waltham, USA) and incubated with Accumax (Merck Life Science) at 37°C for 4−5 min to enable cell detachment. Cells were incubated for an additional 5 min at 4°C following the addition of cold SyncroPatch recording solution (see below) to allow for membrane recovery prior to manual agitation and removal of cells from the flask. Cells were centrifuged for 5 min at 250 g, the supernatant removed and resuspended in divalent free SyncroPatch solution (see below) to a density of 250−500 000 cells/ml. The cellular suspension was incubated for an additional 30−60 min at 4°C and finally transferred to the shaking SyncroPatch cell platform, which was maintained at 10°C throughout the experiment.

### (b) High throughput patch-clamp set-up and solutions

To get the highest quality recordings, it is important to note that we used fluoride-free plates and solutions [34], as in the first attempts in fluoride-containing pilot experiments, we saw larger nonlinear time-dependent leak currents that were difficult to isolate and remove [15].

Patch-clamp experiments were performed on the SyncroPatch 384PE (Nanion, Munich, Germany). Nanion, 1 hole, medR FF (fluoride free, 4−4.5 MΩ) SyncroPatch plates were used to run 384 whole-cell, patch-clamp recordings in parallel. Cell catching, sealing, whole-cell breakthrough and capacitance compensation procedures were automated by the SyncroPatch. According to Nanion’s fluoride-free chip procedures, fluoride-free experimental plates were pre-treated with 0.5 mM NaOH, and washed three times with water and divalent free solution as part of the automated programme. In addition, to improve the success rate with respect to series resistance, the membrane perforator, Escin (15 μM, Merck Life Science) was added to the internal solution and washed out with Escin-free internal solution following the whole-cell pressure pulse step. Experiments were performed at ambient temperature (25 ±1°C for our SyncroPatch at stable operating temperature [28]). Ambient temperature recordings were performed here to improve the success rate of the experiment. The resultant models were adjustable post hoc (e.g. with Q_10_ scalings) for use at physiological temperatures; however, refitting the kinetic parameters to higher temperature data may be a more suitable approach [35].

The fluoride-free internal solution [15] contained, 120 K gluconate, 10 mM KCl, 10 nM NaCl, 10 mM HEPES and 5 mM EGTA, and adjusted to pH 7.2 with KOH. The divalent free solution used for the cell suspension and initial filling of the SyncroPatch plates contained, 140 mM NaCl, 4 mM KCl, 5 mM glucose and 10 mM HEPES for all experiments. Seal-enhancing solutions employed to improve cell-to-plate seal performance contained, 140 mM NMDG-Cl, 4 mM KCl, 4 mM CaCl_2_, 1 mM MgCl_2_, 5 mM glucose and 10 mM HEPES. The recording solution contained, 80 mM NaCl, 60 mM NMDG-Cl, 4 mM KCl, 2 mM CaCl_2_, 1 mM MgCl_2_, 5 mM glucose and 10 mM HEPES. All solutions, except for internal, were adjusted to pH 7.4 with NaOH. All chemicals, unless otherwise stated, were purchased from Merck Life Science. Liquid junction potential was calculated and adjusted in SyncroPatch protocols accordingly.

### (c) Pharmacological isolation of I_Kr_ current

**Figure 4.**
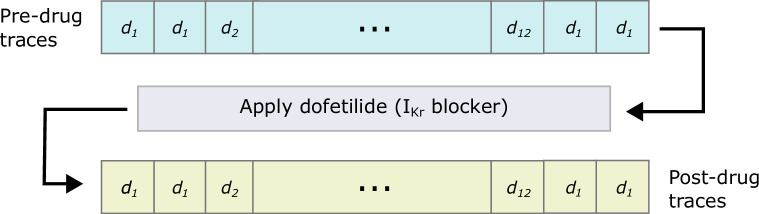
The order in which the protocols were performed. First two sweeps of $d_{1}$ (the *staircase* protocol) are performed, then a single sweep of each of the other protocols are performed before two final sweeps of $d_{1}$. Then, after the addition of 1μM dofetilide (which should provide a specific I_Kr_ block), this sequence of protocols is repeated once more. This allows the subtraction of post-drug traces from pre-drug traces, which mitigates the presence of endogenous (non-I_Kr_) currents and any other artefacts.

After applying each voltage protocol to our cells, we add *dofetilide* at 1 μM (a concentration known to almost fully block I_Kr_) and repeat each protocol in the same order as shown in figure 4. By performing leak correction and subtracting the post-drug leak-corrected trace from the pre-drug leak-corrected trace, we are able to isolate I_Kr_ with minimal contamination from any endogenous or other currents. These postprocessing methods are explained in §5.

Dofetilide (Merck Life Science) was prepared as 10 mM stocks in 100% DMSO. Drug stocks were used immediately or stored at −20°C in glass opaque vials, in small aliquots for single use only (Merck Life Science). In the latter case, stocks were thawed immediately prior to experiments, vortexed and prepared in recording solution to the appropriate concentrations in glass vials. Drug solutions were then transferred to Teflon SyncroPatch plates for automated addition to the SyncroPatch plate following the recording of hERG channel currents in drug-free recording solutions.

## 4. Design of voltage-clamp protocols

A range of *information-rich* voltage protocols were applied sequentially to each well (figure 3). The differences lie in the specified ‘command voltage’, $V_{\text{cmd}}$, that is, the voltage the amplifier is instructed to clamp the membrane potential to at each time point during the experiment. These protocols were developed using a range of techniques, as detailed in Lei *et al.* [36]. We briefly describe the rationales for their designs again here.

Of particular importance is the *staircase* protocol [14], $d_{1}$, of which we perform four repeats. These repetitions allow us to ensure that the cell’s response to $V_{\text{cmd}}(t)$ remains constant over the course of the experiment (see electronic supplementary material, section B). The remaining protocols are performed once each.

Some protocols were designed via numerical optimization with respect to various objective functions. In particular: protocols $d_{3},d_{8}$ and $d_{9}$ were found using the *space-filling curves* approach described in [37]; protocols $d_{10}$ and $d_{12}$ were found using Sobol sensitivities [38] of the Wang and Beattie models, respectively; protocols $d_{4}$ and $d_{5}$ were found using a brute-force approach to maximize the sensitivity of model output to changes in parameters for the Beattie and Wang models, respectively; protocol $d_{7}$ was found by considering 3-step blocks, randomizing the durations of each step and optimizing the voltages; whereas, protocol $d_{11}$ was found by randomizing the voltages and optimizing the durations.

The remaining three protocols ($d_{1},d_{2}$ and $d_{6}$) were designed manually, without the use of an algorithm. The Lei *et al. staircase* protocol, $d_{1}$ was shown to permit the estimation of transition-rate parameters in models of I_Kr_ [14]. A similar protocol, $d_{2}$ is included because it includes a new central section in which there are more short-duration segments. We expect that this protocol highlights more I_Kr_ short-timescale behaviour (namely its *inactivation*/*recovery-from-inactivation* processes, which occurs very rapidly). Finally, $d_{6}$ is performed without the intention of providing useful parameter estimates—in fact, our models are *practically unidentifiable* under $d_{6}$ [12]. Nevertheless, this protocol consists of a sequence of action-potential voltage traces and, hence, provides physiologically relevant data for model validation.

The protocols described above were performed sequentially before and after the addition of dofetilide, a drug considered to block I_Kr_ specifically at this concentration [39]. For quality control (QC), we perform four repeats of the *staircase* protocol, $d_{1}$; twice at the beginning of the experiment and twice after all other protocols. This allows us to discard data from wells that do not remain stable over the course of the experiment (see electronic supplementary material, section B). All other protocols were performed exactly once before, and once after, the addition of dofetilide. This procedure is illustrated by the schematic in figure 4.

Each protocol contains some common elements which are included to aid postprocessing, as described in the following section. In particular, each protocol includes an identical section at the beginning of the protocol (the *leak* ramp). These sections allow for the estimation of leak-model parameters, and to infer the *reversal potential*, $E_{\text{Kr}}$. Each protocol begins at the *holding potential*, $V_{\text{cmd}}(0)=-80\;\text{mV}$, before $V_{\text{cmd}}$ steps down to −120mV before gradually increasing back to −80mV. Because I_Kr_ is small in this range of voltages ([−120,−80]), this allows the determination of the leak current, equation (2.7). A subsequent segment where $V_{\text{cmd}}$ is held at +40mV permits validation of this leak model by checking that $I_{\text{Kr}}=I_{\text{obs}}-I_{\text{L}}$ is positive during this step (as we would expect [11]). Similarly, a *reversal ramp* section is included at the end of each protocol, the apparent reversal potential of the channel, (that is, $E_{\text{Kr}}$ in equation (2.2)) to be observed. This section begins with a +40mV preconditioning step, before $V_{\text{cmd}}$ is rapidly reduced from −70mV to −110mV. The postprocessing methods applied to the leak-ramp and reversal-ramp sections of our protocols are described in the following section.

## 5. Fitting mathematical models to patch-clamp data

### (a) Postprocessing

#### (i) Leak-model fitting

The leak ramp at the beginning of each voltage protocol, and the reversal ramp at the end of each voltage protocol, aids our postprocessing. We use the leak ramp to fit a linear leak-current model to the data, allowing us to subtract leak current and observe the remaining current (which is dominated by I_Kr_). Details of this *leak-correction* procedure are provided in the electronic supplementary material, section A. As mentioned in §3, we also perform *drug subtraction*, whereby our protocols are repeated after the addition of dofetilide, a known I_Kr_ blocker (after which we assume the maximal conductance, g=0 and so $I_{\text{Kr}}=0$). This should minimize the presence of any endogenous (that is, non-I_Kr_) currents in our postprocessed traces. We use this leak-corrected and drug-subtracted data for model fitting and validation

The results of our leak-correction, drug subtraction and reversal potential inference are also used for QC. The QC criteria we use to select wells largely follow those of [28], which are, where possible, applied to all protocols. Though, we also include QC criteria involving $E_{\text{obs}}$ and the relative sizes of the post-drug leak-corrected trace and the pre-drug leak-corrected traces. These criteria result in the removal of most wells from consideration, but those remaining exhibit clean signals (low noise) and great consistency over the course of the experiment (both in terms of the current recorded during the *staircase* protocol, and during the reversal ramps of each protocol). As a result of these criteria, we consider only the data from eight of the 384 wells present. Full details regarding our postprocessing and QC procedures are provided in the electronic supplementary material, sections A and B.

#### (ii) Reversal potential inference

We infer $E_{\text{Kr}}$ from the data using the reversal-ramp segment [28] at the end of each protocol. Following leak correction and drug subtraction, we estimate the reversal potential by fitting an order−4 polynomial to the current and use this to identify the time, $t^{*}$ at which $I_{\text{Kr}}(t^{*})=0$. We let $E_{\text{obs}}=V_{\text{cmd}}(t^{*})$ denote the *observed* reversal potential. To account for discrepancy between $E_{\text{Nernst}}$ and $E_{\text{obs}}$, we assume that any difference in these values is due to some voltage offset, that is, $V_{\text{off}}=E_{\text{Nernst}}-E_{\text{obs}}$. This discrepancy and some alternative approaches are discussed in the electronic supplementary material, section D.

### (b) Computational methods for model fitting

#### (i) Forward simulation of Markov models

As introduced in §2, each of the four Markov models we employ may be seen as systems of ODEs. When $V_{\text{m}}$ is held constant, our governing equation is a linear system of ODEs, for which there exists a range of computational methods [40]. However, the same is not true during the leak ramp and reversal ramp, where the solution cannot be expressed as a matrix exponential, and we instead resort to numerical integration methods. In particular, we use LSODA [41], an algorithm designed for the solution of stiff ODE systems. Here, we set both the relative and absolute tolerances to $10^{-8}$ to ensure the accuracy of our solutions.

#### (ii) Optimization

We fit our models to time-series data by computing MLEs under the assumption that our observations are subject to IID, additive Gaussian noise. That is, for a given protocol, d, we compute


(5.1) $${\hat{\theta }}_{d}={\text{argmin}}_{\theta }\left\{\sum_{i=1}^{n_{d}}{\left(y_{i}(\theta ;d)-z_{i}\right)}^{2}\right\},$$

where $n_{d}$ is the number of observations in protocol d, our i^th^ observation is denoted by $z_{i}$ and the model output (for the i^th^ observation) for a given parameter vector θ is denoted by $y_{i}(\theta ;d)$. To fit our models, we seek a solution to this optimization problem, equation (5.1). A general closed-form solution is not available, so we must resort to numerical optimization methods. We have shown previously that such a model (and computational methods) permit accurate parameter estimation [24].

To fit our models, we use CMA-ES [42], a stochastic optimization method which proposes improved parameter estimates according to some continually updated sampling distribution, providing a chance to escape from local optima. Moreover, we repeat our optimization 30 times from different initial sampling distributions, which allows us to explore more of the model’s parameter space and, provided we reliably recover the same parameter estimate, demonstrates that we are able to identify the true global optimum (that is, we can reliably compute equation (5.1)). For all models except the Wang model, we select the population size (that is, the number of parameter vectors sampled for each generation) by computing the integer


(5.2) $$\begin{array}{lcr} & n_{\text{pop}}:=\left\lfloor 4+3\;\ln(n_{\text{p}})\right\rfloor , & \end{array}$$

where $n_{\text{p}}$ is the number of model parameters, using the heuristic suggested by the PINTS package [43]. In the case of the Wang model, we instead increase the population size to 50. Open source code is publicly available, please see ‘Data accessibility’ at the end of the manuscript.

#### (iii) Sampling of initial guesses

Following [24], we perform each optimization multiple times using different initial guesses. Here, we use 30 initial guesses for each sweep of each protocol. As in [24], our initial guesses for our ‘A’ and ‘b’ parameters in $Q_{i,j}=A\;\exp\{\pm bV\}$ rates are sampled from a log-uniform distribution. In particular, for each transition-rate parameter, p (A or b parameters), we say its logarithm is uniformly distributed such that $\log_{10}(p)∼U(-7,1)$. Similarly, initial guesses of the maximal conductance parameter, g, are generated using the same log-uniform distribution. If a parameter set fails to satisfy the above transition-rate constraints, it is discarded and we resample.

#### (iv) Parameter space boundaries

We constrain our parameter space to mitigate the stiffness of our model’s governing equation (equation (2.1)). In particular, following [13], we ensure that the maximum value obtained by each transition rate $Q_{i,j}=A\;\exp\{\pm bV\}$ (for voltages in the range [−120mV,+60mV]) satisfies


(5.3) $$1.67\times 10^{-5}\;{\text{ms}}^{-1}⩽k_{\text{max}}⩽1\times 10^{5}\;{\text{ms}}^{-1}.$$

We also apply lenient bounds on the maximal conductance, g, using the maximum current observed during the −120mV step at the beginning of each protocol. Further details regarding sampling of initial guesses and boundaries are provided in the electronic supplementary material, section C.

### (c) Results of model fitting and validation

#### (i) Optimization results

**Figure 5.**
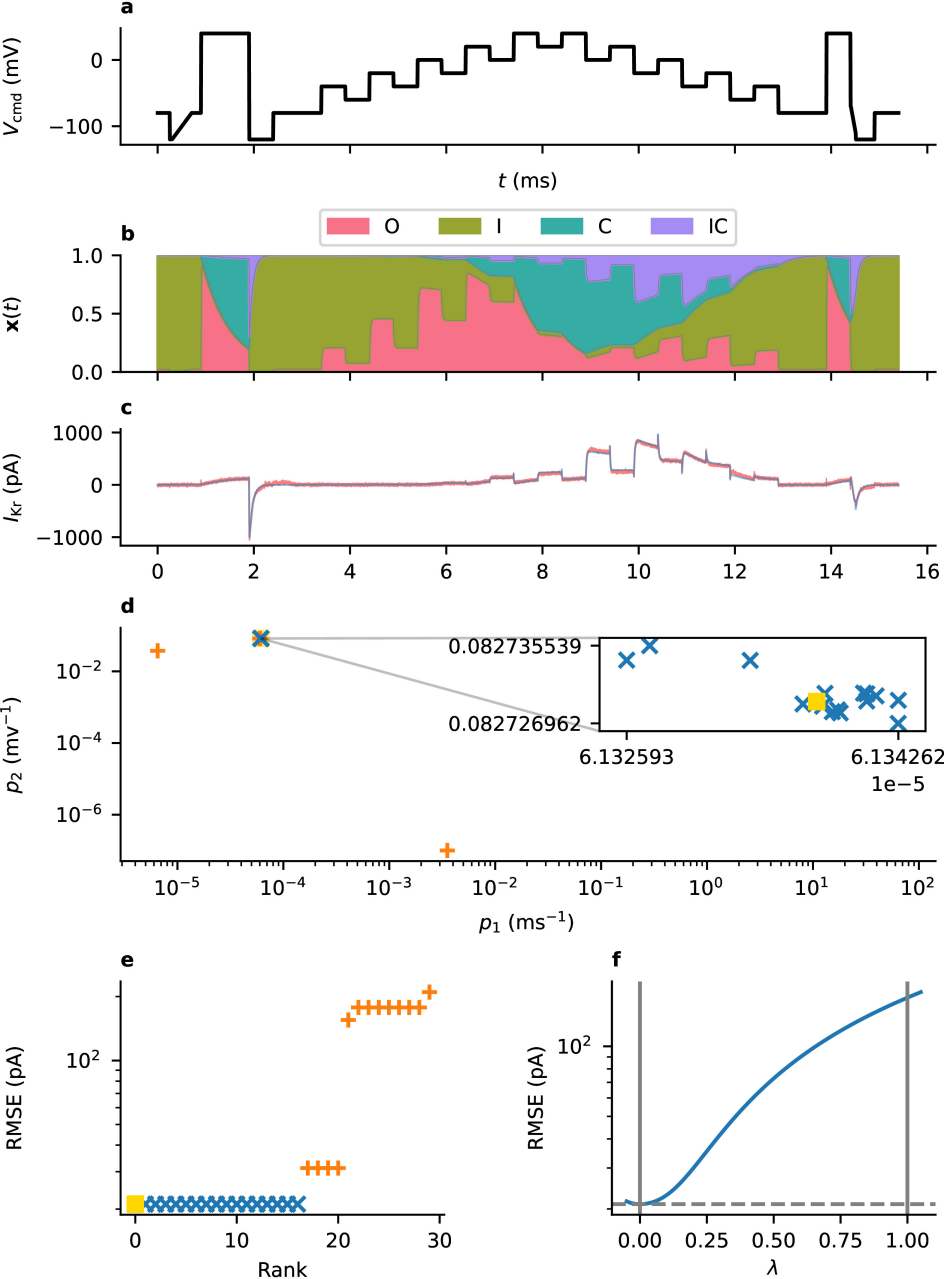
The Beattie model is fitted to time-series data (taken from Well B20 using protocol $d_{1}$) using 30 repeated runs of a stochastic optimization method (CMA-ES) from randomized starting points. (a) The protocol used for data collection ($d_{1}$, the staircase protocol). (b) The occupancy of each state of the Beattie model over the course of the protocol according to the fitted parameter set. (c) The current recorded under this protocol (red) and best model fit (blue). (d) The values found for two transition-rate parameters, $p_{1}$ and $p_{2}$, from multiple optimization runs. The blue markers shown in the inset correspond to those results where the RMSE is at most 101% of the minimum value found. (e) The RMSE error for each parameter set. (f) A cross-section through the likelihood surface, starting at our best estimate of the parameters (λ=0, yellow square) and finishing (when λ=1) at a parameter set with identical maximal conductance (g), but where the transition-rate parameters are taken from the model's original publication (Cell 5) [11].

To be confident that we have successfully identified the optimal parameter set, we should expect that we obtain similar results from repeated runs of our optimization procedure (which both starts from a randomized initial guess, and is inherently stochastic). Figure 5 shows the results of one particular optimization task with 30 repeats. Here, we can see that among our best runs, the resulting parameter set varies only slightly—those results which correspond to a less than a 1% increase in root-mean-square error (RMSE) when compared to the best found parameter set. In this case, these parameter sets occupy a small region of parameter space, suggesting (though not guaranteeing) that our optimization methods are able to reliably find the global optimum.

#### (ii) Quantifying predictive accuracy

**Figure 6.**
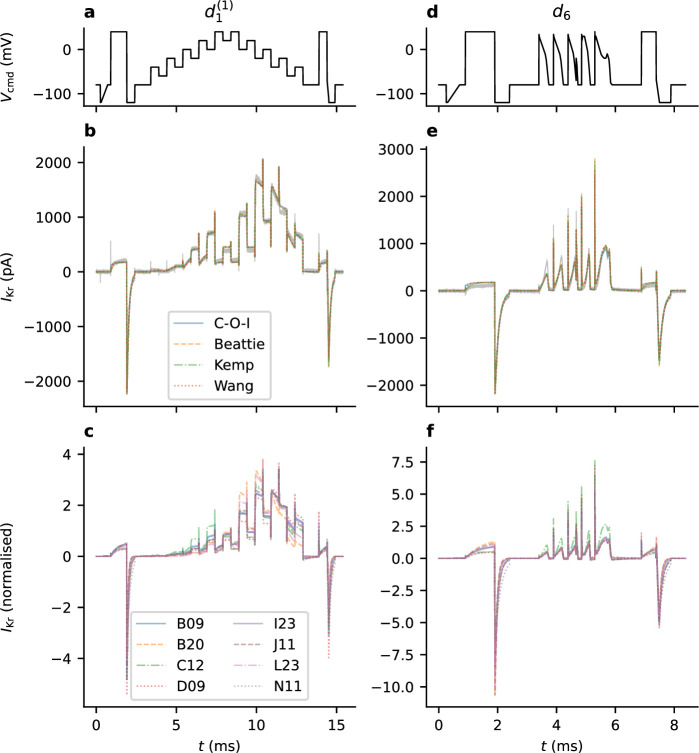
The dependence of model predictions on the particular model structure and wells used for model fitting. All panels on the left relate to sweep 1 of the *staircase* protocol, $d_{1}^{\left(1\right)}$: (*a*), $d_{1}$ protocol; (*b*), fits obtained using each of our models for one particular well, C12; and (*c*), fits obtained using the Beattie Model for each well. Similarly, all panels on the right relate to our validation protocol $d_{6}$: (*d*), the $d_{6}$ protocol; (*e*), predictions made using parameter estimates obtained from the fits shown in (*b*), using each model structure, for the same well, C12; and (*f*) Beattie model predictions of $d_{6}$ using parameter sets obtained from multiple wells (fits shown in (*c*)). The model output shown in (c) and (f) is normalized such that the RMSE of each trace is 1.

Not only do our models provide a good fit to the data (as exemplified in figure 5), but the same parameter estimates perform well when predicting recordings under other protocols. This is largely true across each of our model structures, as demonstrated for predictions of protocol $d_{6}$ currents in figure 6 (panel **e**). Additionally, there is notable variability in the parameter estimates obtained from different wells (using the same fitting protocol). This is shown in figure 6 (panels **c** and **f**) where we see differences in (normalized current) fits and predictions across different wells.

**Figure 7.**
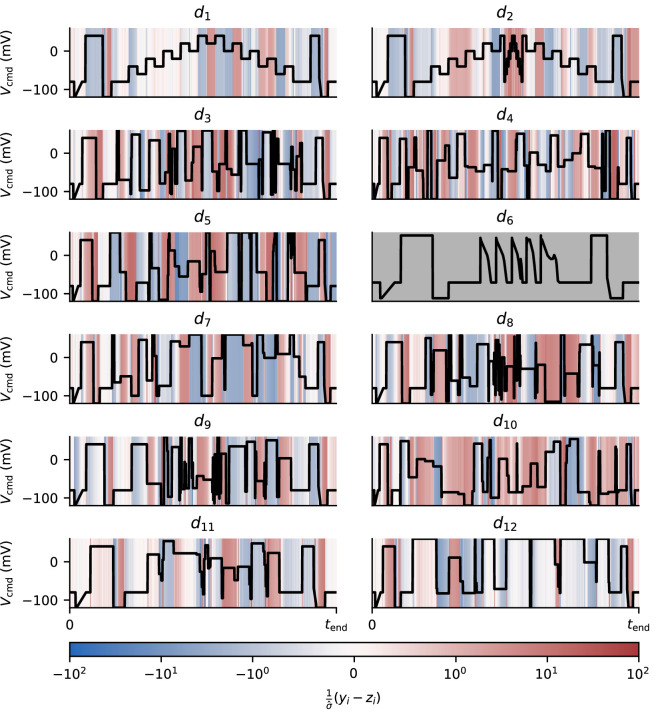
Residuals obtained when fitting the Beattie model to each training protocol (averaged across wells). The values plotted for $\frac{1}{\hat{\sigma }}(y_{i}-z_{i})$ have been clipped to lie between −100 and +100. Protocol $d_{6}$, which is used only for validation and not fitting, is shown in grey.

From each of our parameter sets (each obtained from a different sweep of the data), we obtain an ensemble of parameter estimates. When used as an input to our mathematical models, these parameter estimates give rise to an ensemble of predictions. In figure 7, we show which sections of our voltage protocols prove particularly difficult to fit by computing a weighted average of the residuals of our fitted models at each time point. This average is weighted according to the size of our noise estimate, that is, we compute, $\frac{y_{i}-z_{i}}{\hat{\sigma }},$ and average this quantity across wells. The resultant, shown in figure 7, highlights the sections of our protocols for which the Beattie model consistently over or underestimates I_Kr_. Here, we see that the Beattie model is unable to fully recapitulate the data where our model fitting leads to the current being consistently overestimated in the central, rapidly stepping portion of $d_{2}$, for example. The other model structures demonstrate similar inadequacies, as shown in the electronic supplementary material, section E.

**Figure 8.**
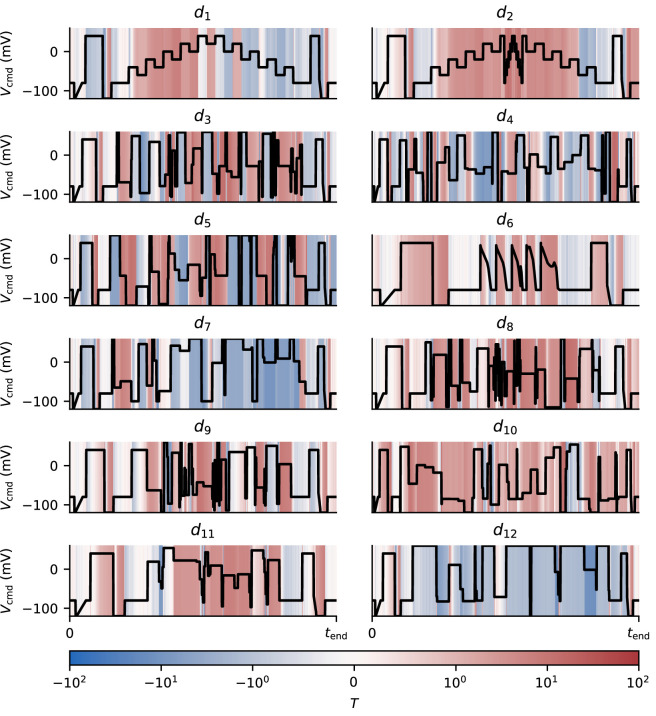
The average behaviour of the Beattie model when producing predictions for unseen protocols, averaged across wells. Sections of the protocols highlighted in red show where the model consistently overestimates I_Kr_, as quantified by T equation (5.4). Whereas, sections highlighted in blue show consistent underestimation. The T values are clipped between −100 and +100.


Similarly, figure 8 shows sections of our validation protocols where the Beattie model (when fitted using the full range of training protocols) consistently over- or under-predicts I_Kr_. Here, we quantify the tendency of a model’s predictions to be consistently discrepant at each time-point, $t_{i}$, by calculating

(5.4) $$\begin{array}{lcr} & T=\frac{{\bar{y}}_{i}-z_{i}}{\left(\hat{\sigma }+\frac{\text{std}(y_{i})}{N_{\text{predictions}}}\right)}, & \end{array}$$

where ${\bar{y}}_{i}$ and $\text{std}(y_{i})$ are the mean and standard deviation, respectively, of our $N_{\text{prediction}}$ model predictions at time $t_{i}$. When T≫0 or T≪0, this statistic shows that our ensemble of predictions consistently makes inaccurate predictions. Moreover, the sign of T indicates where models tend to over or underestimate I_Kr_. Here, we only include predictions and not model fits—that is, we discard parameter estimates obtained from the protocol under consideration. In contrast to figure 7, we see that the Beattie model (under various fitted parameter sets) seems to consistently over or underpredict the current during certain protocols—during protocols $d_{2},d_{8}$ and $d_{10}$, for example, we see a consistent overestimation of the current. This is indicative of the protocol-to-protocol variability we expect to find when fitting discrepant models [24]. Similar figures demonstrating the tendency for each model structure to produce over or underpredictions for certain sections of each protocol are shown in the electronic supplementary material, section E.

To compare accuracy of model predictions across different cells we use the *normalized* RMSE (NRMSE),


(5.5) $$\begin{array}{rl} \text{NRMSE}(y,z):= & \frac{\text{RMSE}(y,z)}{\left\|z\right\|}=\frac{\sqrt{\sum_{i=1}^{N_{\text{obs}}}(z_{i}-y_{i}{)}^{2}}}{\left\|z\right\|}, \end{array}$$

where RMSE denotes the (non-normalized) root mean square error. As the maximal conductances of different cells may vary, NRMSE is a way to normalise for current magnitude, to compare and average predictive performance across wells fairly. It also allows us to compare protocols with different numbers of observations.

To compare the average fitting and predictive accuracy of these models (across all wells and protocols), we introduce


(5.6) $$E_{\text{predict}}=\frac{1}{(N_{\text{d}}-1{)}^{2}}\sum_{d\in D\setminus \{d_{6}\}}\left\{\sum_{\tilde{d}\in D\setminus \{d\}}\text{NRMSE}\left(I_{\text{Kr}}(\theta_{d};\;\tilde{d}),z_{\tilde{d}}\right)\right\},$$

where D is our full collection of *N*_*d*_ protocols, $\theta_{d}$ is the parameter estimate obtained from protocol d, the vector, $I_{\text{Kr}}(\theta_{d};\tilde{d})$ is the model output under protocol $\tilde{d}$ obtained using the parameter estimate $\theta_{d}$, and $z_{\tilde{d}}$ are the data obtained under protocol $\tilde{d}$. The first sum excludes the action potentials protocol (*d*_*6*_) which was not used for fitting, due to low identifiability of parameters. The second sum excludes terms where the fitting and validation protocol are identical (terms for which $d=\tilde{d}$). As such, because of the diverse nature of our set of protocols, this sum describes the performance accuracy of the model in as-yet-unseen situations. Additionally, we compute


(5.7) $$E_{\text{fit}}=\frac{1}{N_{\text{d}}-1}\sum_{d\in D\setminus \{d_{6}\}}\text{NRMSE}\left(I_{\text{Kr}}(\theta_{d};d),z_{d}\right).$$

Equation (5.7) is an average over the RMSE between our fits and the data (down-weighted by the magnitude of the recorded trace). Where there are multiple repeats of a protocol (as for our four repeats of $d_{1}$), we treat these values as separate protocols in equations (5.6) and (5.7).

**Figure 9.**
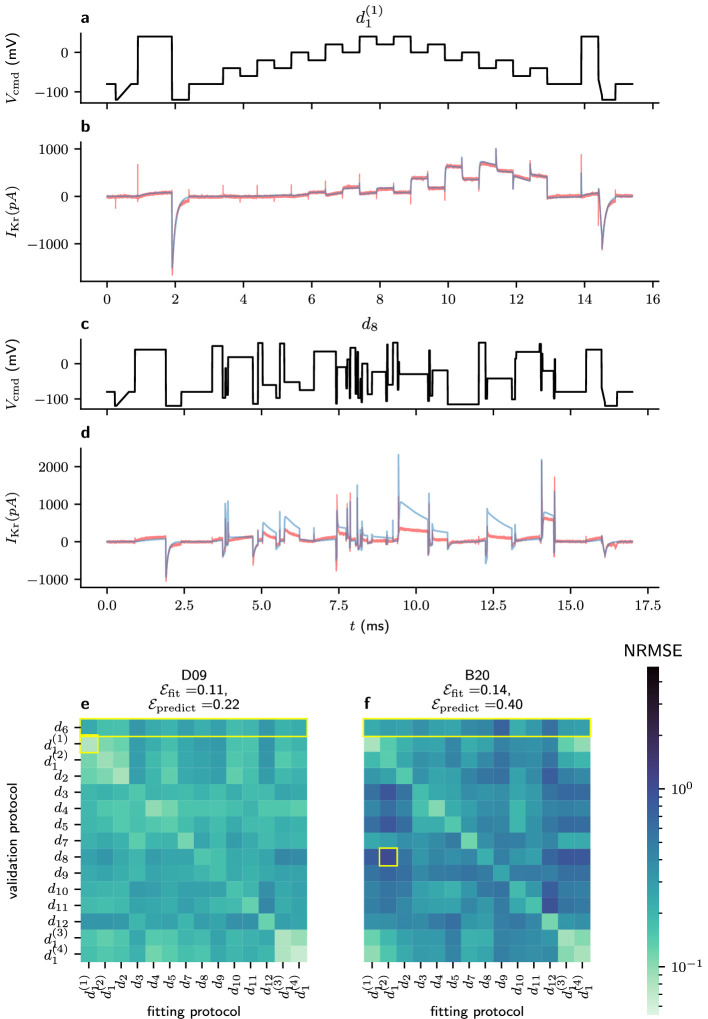
A cross-validation heatmap showing the predictive accuracy of the Beattie Model. (*a*) and (b) show a comparison between the data (red) and the current fitted using the Beattie model from Well D09 (corresponding to the highlighted square in (e). Similarly, (c) and (d) show the $d_{8}$ protocol and the worst Beattie Model prediction from Well B20 (corresponding to the highlighted square in (f). (e) and (f) show cross-validation heatmaps for Wells D09 and B20, respectively, which are the wells with the lowest and highest average NRMSE across all pairs of fitting and validation protocols.

The accuracy of these model predictions (indexed by the sweeps used for fitting and those used for validation) may be summarized in a heatmap, as shown in figure 9. This shows the accuracy of each member of our ensemble of predictions for two particular wells (Well D09 and Well B20), and a particular choice of model structure (the Beattie model [11]). Then, we take the average of these values and show (for each candidate model structure) the average predictive accuracy under each pair of fitting and validation protocols. Here, the worst prediction shown (in figure 9*d*) shows a consistent underestimation of the current during the application of protocol $d_{8}$. Here, the Beattie model parameters obtained to $d_{1}$ (the *staircase* protocol) seem unsuitable for predicting protocol $d_{8}$ (and, in the case of Well B20, all protocols except $d_{1}$ and $d_{2}$). As this model seems to be practically identifiable under protocol $d_{1}$ [24], this lack of predictive accuracy when the model is fitted to protocol $d_{1}$ (particularly in the case of Well B20) suggests model discrepancy.

**Figure 10.**
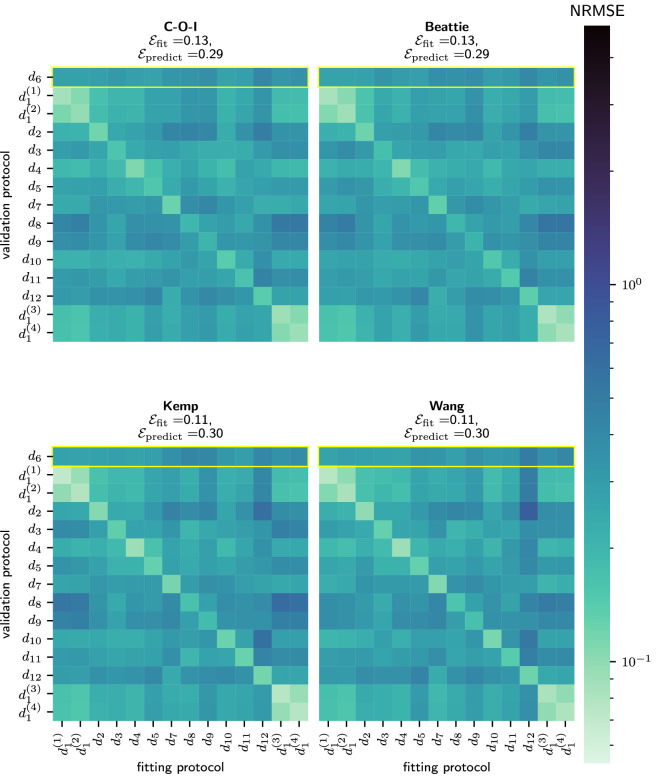
A comparison of the predictive performance of our chosen model structures. Each heatmap shows the average normalized RMSE when the given model is fitted and validated each pair of protocols. Here, the diagonals show the NRMSE obtained during model fitting ($E_{\text{fit}}$), which, in every case, is noticeably lower than the error in any of the corresponding predictions ($E_{\text{predict}}$).

Figure 10 compares each model’s averaged, cross-validation heatmap. Here, we see that the simpler model structures with fewer states and parameters (namely, the C-O-I model and the Beattie model) seem to produce less accurate fits, but more accurate predictions when compared to the more complex models (the Kemp and Wang models). It is noteworthy, however, that the poor predictive accuracy of models fitted to protocol $d_{1}$ is less apparent for the Wang model than for other model structures.

## 6. Variability in parameter estimates

By fitting our data to each sweep in our dataset, we obtain a collection of parameter estimates for each model structure, where each individual parameter-estimate vector, $\hat{\theta }$, pertains to a particular sweep in our dataset. For each Markov model, and for each of the eight wells selected by QC, there are 14 parameter-estimate vectors—each arising from each repeat of each fitting protocol (a single repeat of each protocol except $d_{6}$, and an additional three repeats of $d_{1}$). In this section, we discuss the variability of these parameter estimates using a simple, linear statistical model.

**Figure 11.**
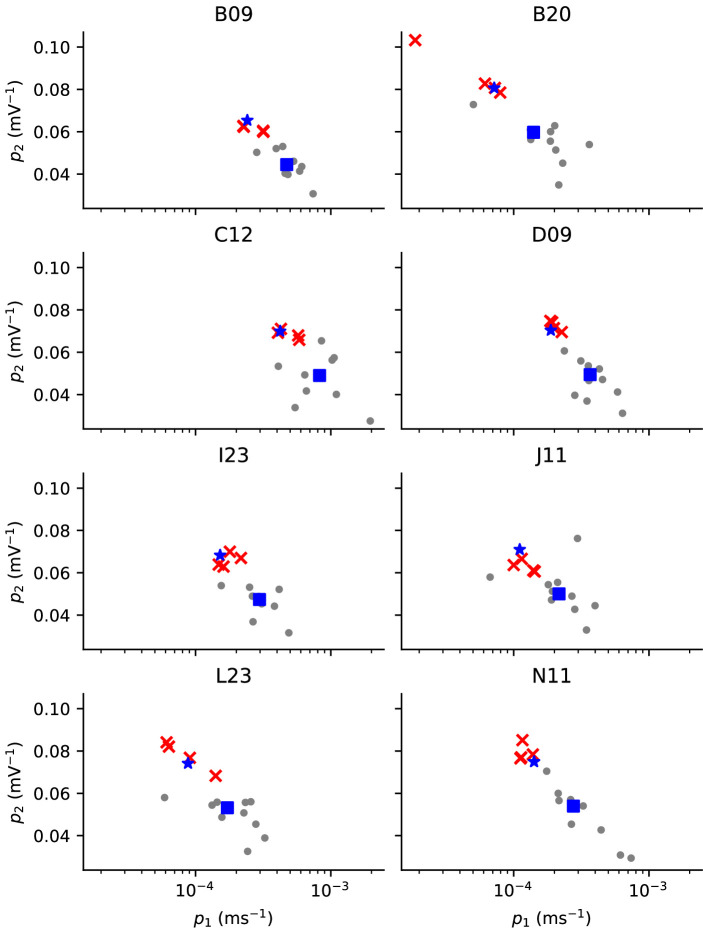
A linear statistical regression model including well- and protocol-dependent effects, $M_{\text{w},\text{d}}$, largely fits and recapitulates the well- and protocol-dependence in parameter estimates for the Beattie model. Each panel shows the same two parameter estimates obtained from a given well, and highlights the parameter estimates obtained from the *staircase* protocol (red crosses). The parameter estimates obtained from the same well, but from a different protocol, are shown by the grey circles in each panel. The blue square shows the well-dependent effect according to $M_{\text{w}}$, our linear model with no protocol-dependent effects (that is, only well-dependent effects), and the blue star shows the sum of the well and protocol effects (for the given well and the $d_{1}$*staircase* protocol) according to the $M_{\text{w},\text{d}}$ model. Note that $p_{1}$ (the *abscissae*) has been log-scaled, as it is in our linear model. Note that only two of the eight model kinetics parameters ($p_{1}$ and $p_{2}$) are shown as an illustration, but the linear model is constructed for all eight.

Plots of our parameter estimates, shown in figure 11, suggest a relationship between the estimate and the well-protocol combination it was obtained from; some protocols yield consistently high estimates of $p_{1}$ when compared to other protocols, for example. These plots (figure 11) show only the model’s first two parameters ($p_{1}$ and $p_{2}$), which determine a single transition rate, $k_{1}=p_{1}\exp\{p_{2}V\}$ in the Beattie model [11] and, as such, do not provide a complete picture of the variability of parameter estimates.

To provide further insight, we fit a simple statistical linear regression model $M_{\text{w},\text{d}}$ for our parameter estimates, including well (w) and protocol/design (d) effects. Where our models contain transition rates of the form, k=Aexp⁡{±bV}=exp⁡{a+bV}, we fit our linear model using a and b. Only parameters related to the transition-rate matrix Q are included in this analysis because we anticipate noticeable cell-cell variability in maximal conductances, whereas we expect transition rates to be closely related to biophysical constants [12]. To quantify the well- and protocol-dependence in our parameter estimates, we use log-likelihood differences (LLDs). We do this by considering


(6.1) $$\begin{array}{rl} M_{\text{w},\text{d}}:\;Y & =\mu +X_{\text{d}}\beta_{\text{d}}+X_{\text{w}}\beta_{\text{w}}+E, \end{array}$$

where: Y is an $N_{\text{trace}}\times N_{\text{p}}$ matrix where $N_{\text{trace}}$ is the number of fitting traces and $N_{\text{p}}$ is the number of parameters in our I_Kr_ model, such that each row is a parameter estimate obtained from model fitting; $X_{\text{w}}$ and $X_{\text{d}}$ are well- and protocol-effect *design matrices* where each row encodes the well/protocol used for a given parameter estimate; $\beta_{\text{w}}$ and $\beta_{\text{d}}$ are our parameter matrices, with each row representing a particular well or protocol effect (respectively), and with each column corresponding to a different parameter in our I_Kr_ model; E is a matrix of random errors, such that for each $k\in \left\{1,\ldots ,N_{\text{p}}\right\}$, the errors in the k^th^ column are IID Gaussian distribution random variables with expectation zero and unknown standard deviation $\sigma_{k}$. So that this model is identifiable, we insist that the protocol and well effects sum to zero, that is, $\beta_{\text{w}}^{⊤}1=0$ and $\beta_{\text{d}}^{⊤}1=0$. The remaining models, $M_{\text{w}}$, $M_{\text{d}}$ and $M_{0}$ are set by the constraints, $\beta_{\text{w}}=0$, $\beta_{\text{d}}=0$ and $\beta_{\text{d}}=\beta_{\text{w}}=0$, respectively. In this way, model $M_{\text{w}}$ includes only the well-dependent effect and assumes that the parameter estimates obtained are subject to IID Gaussian random errors, but are independent of the particular protocol used to fit them. Similarly, model $M_{\text{d}}$ includes only the protocol-dependent effect and not the well-dependent effect. Finally, model $M_{0}$ assumes that there is no well- or protocol-dependence, but that the variability in our parameter estimates is solely due to IID Gaussian errors. The suitability of the full model, $M_{\text{w, d}}$ is demonstrated in figure 11, which shows the protocol-dependent effect corresponding to protocol $d_{1}$. This linear model suggests that the differences between parameter estimates obtained from different wells and different protocols can be explained by simple translations in the parameter space (that is, two translations which independently describe the protocol-dependent effect and the well-dependent effect).

We list the maximum likelihood of each model, $M_{i}$, for each set of parameter estimates (arising from our candidate I_Kr_ model structures) in table 1, as well as the resulting LLD between the full model $M_{\text{w,d}}$ and the model without well effects $M_{\text{d}}$ (**LLD(-w)**), and $M_{\text{w,d}}$ and the model without design/protocol effects $M_{\text{w}}$ (**LLD(-d)**). Thus, **LLD(-w)** is a statistic quantifying the size of the well-dependent effects, and **LLD(-d)** is a statistic quantifying the size of the design/protocol-dependent effects. From the values listed in table 1, we can see that including the well-dependent and protocol-dependent effects leads to a large increase in likelihood. This indicates that there is significant protocol- and well-dependence in our parameter estimates. The parameter estimates that we obtained are shown in figure 11. From these results, we can see that there is noticeable variability between parameters obtained from different wells under the same protocol, and also variability between the estimates taken from the same protocol, but from different wells.

**Table 1.** Log-likelihoods and likelihood ratios (LLDs) for each of the linear regression models, applied to all of our biophysical models (that is, the collection of parameter estimates obtained using each biophysical model). Here, we see that in each case, and for each biophysical model, that both well- and protocol-effects are very significant. While **LLD(-d)**, the log likelihood difference between full model (with both protocol/design ‘d’ and well ‘w’ effects) and just well effects, suggests that there is discrepancy between the recordings of I_Kr_ (taken from each well) and the dynamics of our biophysical models, and these are highlighted by the different protocols. The large magnitude of **LLD(-w)** (across all model structures) suggests the presence of latent, well-dependent effects. The larger of these two values is highlighted in bold for each row.

Model	$M_{0}$	$M_{\text{w}}$	$M_{\text{d}}$	$M_{\text{w},\text{d}}$	LLD(-w)	LLD(-d)
C-O-I	1465.6	1668.5	1724.4	2111.1	386.7	442.6
Beattie	1563.3	1817.0	1784.2	2247.2	462.9	430.2
Kemp	931.8	1099.8	1113.7	1356.3	242.6	256.5
Wang	−2082.1	−1976.1	−1836.7	−1673.5	163.2	302.6

## 7. Discussion

We have collected new data using multiple information-rich experimental designs and used these to thoroughly validate our models of I_Kr_. The methods used to process these data, including our fully automated QC procedure, are suitable for future work involving the collection and analysis of multiprotocol patch-clamp experiments for I_Kr_, and possibly other ion-channel currents. Note that we apply far stricter QC than most uses of high-throughout patch clamp (electronic supplementary material, section B). This is because we fit directly to the postprocessed time series, rather than extracting peaks or time constants as is more common in screening settings, and correspondingly the whole trace needs to be accurate. Note, that while we observed a low success rate, the data retained after QC is of very high quality and shows remarkable fidelity to our mathematical models. Even so, future improvements to the experimental equipment and methodology may result in an increased success rate and, perhaps, even cleaner data.

Because our data were collected from a diverse range of information-rich experiments, we are able to thoroughly validate the predictive accuracy of a small selection of I_Kr_ models. Overall, we have shown that each model is able to accurately recapitulate our I_Kr_ recordings. This is particularly true in certain wells, such as Well D09, where our models not only provide very accurate fits to the data, but are able to predict the current during unseen protocols to a high degree of accuracy (see figure 10). Broadly speaking, models with more parameters (the Kemp [31] and Wang [30] models) produced more accurate fits to our data, as quantified by $E_{\text{fit}}$, whereas the simpler models (the C-O-I and Beattie [11] models) produced slightly more accurate model predictions for unseen protocols (see figure 10).

Nevertheless, the difference between our competing model structures regarding $E_{\text{fit}}$ and $E_{\text{pred}}$ are rather subtle. Perhaps our ideal patch assumptions equations (2.5) and (2.6) result in models too discrepant to allow such differences in model structure to make a material difference in these values. If these ideal patch assumptions are relaxed (for example, by the inclusion of experimental artefacts [26]), the data herein should prove valuable for the training and validation of further I_Kr_ models. Our work is ongoing in this regard.

While predictions for a single protocol have been used for model validation before [11], our method provides a more thorough validation of model predictions under a wide range of voltage-clamp protocols. This approach also incorporates the protocol-dependence of parameter estimates, whereby a discrepant model may produce accurate predictions when fitted using a certain protocol, but inaccurate predictions when fitted using another, even when each parameter estimate is practically identifiable. However, as in previous work [11], Markov chain Monte Carlo methods may provide further insight into the role of parameter identifiability [44,45].

Our statistical analysis of our ensembles of parameter estimates suggest that there are strong well- and protocol-dependent effects acting upon our parameter estimates. As discussed in Shuttleworth *et al.* [24], we expect to see these protocol dependent effects when there is discrepancy between our mathematical models and the underlying biophysical mechanisms that we observe. The strong well-dependent effects, however, suggest substantial experimental variability, unaccounted for by the models presented here. As argued by Lei *et al.* [26], it is possible that well-dependent *experimental artefacts* are the dominant cause of this well-to-well variability. Perhaps the large well-to-well variability in our kinetic parameters is caused by the overfitting of kinetic parameters when these effects are omitted. Further work will investigate whether the inclusion of such artefact effects decreases this well-to-well variability, and improves accuracy of our model predictions. If so, the inclusion of artefact effects may result in yet more accurate predictive models of I_Kr_ and allow us to better discriminate between competing model structures on the basis of predictive accuracy and the protocol-dependence of parameter estimates.

## Data Availability

Open source code for all the model fitting and plots in this paper can be found in [46]. A permanently archived version used for this paper is available on Zenodo [47]. Patch clamp data are available from Figshare [48]. Supplementary material is available online [49].
